# Evaluation of Intra-Tumoral Vascularization in Hepatocellular Carcinomas

**DOI:** 10.3389/fmed.2020.584250

**Published:** 2020-10-27

**Authors:** Qi Zhang, Jiajun Wu, Xueli Bai, Tingbo Liang

**Affiliations:** ^1^Department of Hepatobiliary and Pancreatic Surgery, The First Affiliated Hospital, Zhejiang University School of Medicine, Hangzhou, China; ^2^Zhejiang Provincial Key Laboratory of Pancreatic Disease, The First Affiliated Hospital, Zhejiang University School of Medicine, Hangzhou, China; ^3^The Innovation Center for the Study of Pancreatic Diseases of Zhejiang Province, Hangzhou, China; ^4^Zhejiang Clinical Research Center of Hepatobiliary and Pancreatic Diseases, Hangzhou, China

**Keywords:** tumor vascularization, liver cancer, microvascular density, vascular pattern, cell marker

## Abstract

Intratumoral neovascularization has intricate effects on tumor growth, metastasis, and treatment. Over the last 30 years, Microvessel density (MVD) has been the standard method for laboratory and clinical evaluation of angiogenesis. Hepatocellular carcinoma (HCC) is a typical hypervascularized tumor, and the predictive value of MVD for prognosis is still controversial. According to previous viewpoints, this has been attributed to the determination of hotspot, counting methods, vascular endothelial markers, and different definitions of high and low vascular density; however, the heterogeneity of tumor angiogenesis patterns should be factored. The breakthroughs in artificial intelligence and algorithm can improve the objectivity and repeatability of MVD measurement, thus saving a lot of manpower. Presently, anti-angiogenesis therapy is the only effective systematic treatment for liver cancer, and the use of imaging technology-assisted MVD measurement is expected to be a reliable index for evaluating the curative effect. MVD in multinodular hepatocellular carcinoma represents a subject area with huge understudied potential, and exploring it might advance our understanding of tumor heterogeneity.

## Introduction

Liver cancer is the fourth leading cause of cancer-related death with a 5-year overall survival rate of 18% globally (1). Hepatocellular carcinoma (HCC) is the most prevalent primary liver cancer, and its incidence is increasing in different populations in recent decades, specifically in Asian countries due to hepatitis B virus infection. Whilst acknowledging the continuous progress in the treatment of HCC, its prognosis remains poor (2). This is attributed to the fact that only a small part of HCC patients are diagnosed in early stages and receive curative treatments like hepatectomy and liver transplantation. However, the recurrence rate after surgical resection is high, and the application of liver transplantation is limited by the shortage of grafts.

Angiogenesis, a process that facilitates oxygen and nutrient delivery to the tumor cells, is the hallmark of cancer (3). Generally, it is believed that the absence of neovascularization causes the size of the solid tumor to remain in a dormant state of only 2–3 mm^3^ (4). Neovascularization supports tumor cell proliferation and provides a pathway for metastasis (5). Therefore, a tumor with rich blood supply is considered to have a growth advantage and early metastasis compared to poorly vascularized tumors. Liver cancer is a hypervascularized tumor, and angiogenesis regulates disease recurrence, progression, and metastasis. Thus, anti-angiogenesis strategies are developed for liver cancer and are particularly attractive. Currently, sorafenib and lenvatinib, two multi-kinase inhibitors with potent anti-angiogenic capacity, are first-line therapy for hepatocellular carcinoma (HCC) that accounts for 80% of primary liver cancer (6, 7). In addition, evaluation of vascularization might be valuable for predicting prognosis and decision-making for clinical practice (8). For instance, postoperative transcatheter arterial chemoembolization is recommended for patients with microvascular invasion of tumor cells (9). The abundance of micro-vessels is associated with poor outcomes; however, the quantification of micro-vessel abundance is challenging and several methods have been developed (10). As a classical and most widely used measurement of angiogenic activity, microvascular density (MVD) has an effective predictive value in the clinical behavior of many kinds of tumors since it was first proposed by Weidner in 1991 (11). Therefore, it is considered that the measurement of angiogenesis in liver cancer tissue might be of great significance for the prognosis and treatment of liver cancer (12). Herein, we review the progress, agreements, and controversies of microvascular quantification in liver cancer, and its roles in the prognosis and treatment of HCC.

## Biological Characteristics of Microvascular Density

Although highly vascularized tumor is supposed to obtain better blood perfusion and more oxygen supply, in practice, the relationship between tumor MVD and local hypoxia is complicated. Vascular endothelial growth factor (VEGF) is a hexose-modified multifunctional protein, which has a specific binding site of hypoxia-inducible factor-1α (HIF-1α), and can act on vascular endothelial cells and induce micro-angiogenesis. In the development of HCC, hypoxia enhances the transcriptional activity of VEGF, and increases the stability of VEGF mRNA in a HIF-1α-dependent manner (13–15). However, high MVD is prone to but does not always represent a satisfactory blood supply of tumors. Because of the compression by overproliferated tumor cells and the immature vascular structure, tumor neovascularization is structurally abnormal with compromised functions, which will still cause acidosis and hypoxia within the tumor (16). Hypoxia enhances the malignant characteristics of tumor cells by inducing epithelial-to-mesenchymal transition and consequently causing drug resistance and metastasis (17, 18). Certainly, reversing microenvironmental hypoxia in tumor by direct delivery of oxygen or improvement of angiogenesis stimulate the effectiveness of chemotherapy and radiotherapy (19–22). Nevertheless, abundant neovascularization in tumor does promote distant metastasis of tumor cells (23). Therefore, precise evaluation of tumor vascularization and methods for regulation of vascularization in HCC are vital for clinical practice.

However, quantification of both vascular abundance and maturity is formidable. Currently, reliable *in vivo* methods to evaluate vascularization of tumors are unavailable, and *ex vivo* methods are based on two-dimensional analysis, which is not the case in real tumors. So far, MVD is still the most applied and accepted index for the measurement of tumor vascularization. Neovascularization in tumors with high MVD has been confirmed to be positively related to distant metastasis and increased number of circulating tumor cells (24–26). However, debate exists about the association between MVD and the differentiation degree of HCC. Studies by Wada et al. and Hisai et al. found that angiopoietin-2 expression was positively correlated with MVD and that MVD of well-differentiated HCCs is significantly lower than that of moderately and poorly differentiated HCCs (14, 27), whereas other reports showed that there was no correlation between the two groups (28). Regarding drug perfusion and distribution, it is not intuitive that tumors with high MVD can exhibit better drug perfusion. Immature vessels and interstitial fluid pressure can severely hinder the penetration and distribution of drugs, although this feature can be also utilized by nanomaterials to boost drug delivery through the enhanced permeability and retention effect (29). In contrast, the elaborate use of anti-angiogenesis strategy might promote tumor vascular normalization and enhance blood perfusion and drug delivery (16, 30, 31).

## Advances in the Methodology of MVD Measurement

The quantitation method of MVD in the tumor was first proposed by Weidner in 1991 (32). This technique means counting the outline of the blood vessel wall stained by routine immunohistochemistry in tissue slide, and eventually obtaining the number of micro-vessels per square millimeter. Briefly, this method begins from scanning the whole section under a low-power microscope field to identify several “hot spots” with the highest blood vessel density, followed by counting single new micro-vessels under a high-power microscope field (33). The obscurity of whether it was a neovascularization was resolved by an agreement between two researchers using a double-ended microscope, and each procedure took 7–10 min. In our experience, the selection of hot spots should be spaced at an appropriate distance and reflects the overall situation of the whole slice. Neovascularization is distinguished if single new micro-vessels, which are defined as any stained epithelial cells (EC) or clusters separated from adjacent blood vessels, with or without lumen or red blood cells, are observed. Blood vessels containing muscle walls and vessels in the tumor sclerosis area are not counted. In this earliest work, Weidner found that the MVD in breast tumors with poor prognosis and metastasis was twice as high as that in breast tumors with good prognosis and no metastasis. Despite being revolutionary, this method has inherent shortcomings. First, the subjective definition of hotspot causes poor repeatability, thus the inter-observer and intra-observer heterogeneity are significant. For example, de Jong and colleagues reported that the average inter-observer coefficient of variation was about 24% (34). Second, the judgment of neovascularization is subjective with no objective parameters for quality control. Last, manual calculation is time-consuming, particularly for tumors with hypervascularization and size larger than 10 cm. In addition, the cut-off values between high and low MVD in different trials ranged from 4 to 106.3. This may be due to the wide range of antibodies, patient groups, treatments, and data interpretations. Therefore, cross-sectional comparisons of cut-off values are meaningless.

**Figure 1:**
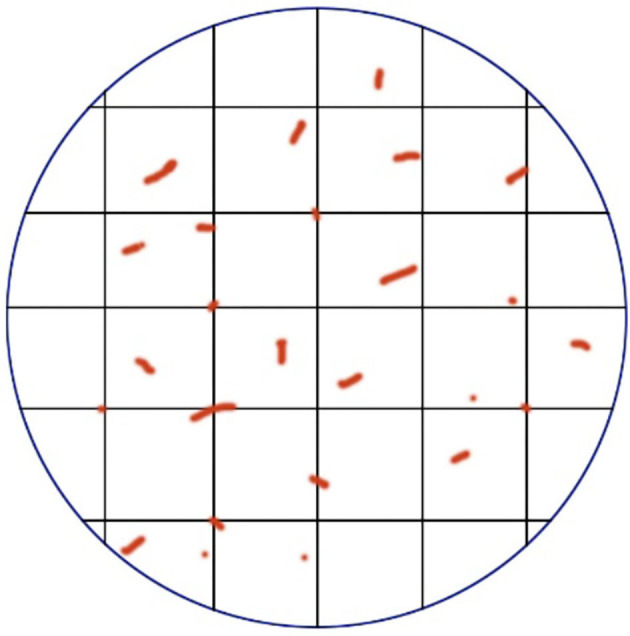
A demonstration diagram of the Chalkley method. The vertical lines that are intersected in the circular eyepiece form a grid, and the vessels were counted when collided with the grid point.

The Chalkley method, also called point-overlap morphometry, is based on the systematic random sampling theory to approximate the relative area of neovascularization in a limited area (35). Like the Weidner method, it requires the subjective selection of several hotspots with the highest neovascularization density, but does not need to calculate all micro-vessels. A special attachment called “Chalkley point array graticule” installed on the eyepiece of the microscope was used (Figure 1), there are 25 randomly distributed fixed points on the Chalkley graticule, and the direction of the grid points can be changed by moving clockwise or counterclockwise (36). The observer can find a unique location where there is the highest grid point overlap with the micro-vessels, and record the total number of intersections as a Chalkley count, where Chalkley count is a unitless parameter. Since there is no need for observers to frequently determine whether the stained microvessels conform to the principles of neovascularization, the Chalkley method is relatively more objective and time-saving than the Weidner method.

To improve the reproducibility of measurement and reduce inter-observer and intra-observer variations, computer-aided technology has been applied to microvessel counting. Unfortunately, no computer software that can automatically analyze and measure MVD is up to date, primarily because manual intervention is necessary to select vascular hotspots. Some investigators have attempted automated image analysis algorithms to generate geographical microvessel maps by calculating entire tumor slices, which can be more objective in counting microvessels (37). Although they conquer the subjectivity of previous methods, the excessive time consumption limits its application, and hence it is worth investigating whether MVD in the whole section represents the real angiogenic activity of the tumor. In recent years, Marien et al. developed a semi-automatic system based on systematic uniform random sampling, i.e., AutoTag and AutoSnap as a substitute for the classical method of identifying hotspots manually by pathologists to confirm areas of interest (ROI). Then, a self-written Photoshop automatically integrates the digital grid with the ROI captured by the system to produce a new image for computer microvascular counting (38). Due to the automatic nature of AutoSnap, workforce hours can be saved, and each image has a tag corresponding to it, making the result traceable. In addition, the random sampling method can reflect the overall vascular growth in the tumor and minimize the variation caused by the experience of the observer at the time of sampling. This technique was further validated by Marien and colleagues in colorectal cancer, glioblastoma multiforme, ovarian carcinoma, and renal cell carcinoma (39), despite being not verified by other investigators. The measurement method of MVD is currently immature and will keep evolving toward being more standard, objective, repeatable, and efficient. With the emergence of computer algorithm and artificial intelligence technology, it is believed that real automatic vascular technology analysis will emerge in the near future. But it must be noted that due to the heterogeneity of tumor vascular growth patterns, certain methods of sampling or measurement might not be suitable for all tumors. Also, the identification of vascular growth patterns and their clinical significance are critical in different types of tumors as well as in different patients with the same types of tumor.

## Endothelial Cell Markers Used to Measure MVD

Many pan-endothelial and special endothelial cell markers are used for vascular labeling in HCC. Among all these markers, the widely used ones include vWF (factor VIII-related antigen), CD31, CD34, and CD105. vWF is a glycoprotein primarily found in endothelial cell Weibel-Palade bodies, and it mediates platelet adhesion to endothelial cells at the sites of vascular injury (40). vWF is probably the most specific endothelial marker, which can well distinguish endothelial cells from other surrounding tissues, due to its exclusive expression on vascular endothelial cells despite a small part of it being stored in megakaryocytes (41). In normal liver, only scattered positive reactions are observed in vascular endothelial cells in the portal area, and vWF is not expressed in normal hepatic sinusoidal endothelial cells. However, positive staining may be found in the background of chronic hepatitis and cirrhosis, whereas strong positive staining is often detected in HCCs (42). These characteristics make vWF one of the most reliable endothelial markers in measuring the MVD of HCC.

CD31, which belongs to the immunoglobulin superfamily, is a component of endothelial cell junction and also appears on the surface of platelets, monocytes, macrophages, plasma cells, neutrophils, as well as other inflammatory cells (43, 44). The expression of CD31 is homogeneous and strongly expressed relative to the vascular type-specific expression of VWF and CD34, and it often cross-reacts with plasma cells (41). However, CD31 is rarely used for MVD study in HCC.

CD34, a transmembrane glycoprotein of 110 kDa, is another endothelial marker broadly used in HCC. Like the vWF and CD31, CD34 is a pan-endothelial marker and is usually located in vascular endothelial cells and hematopoietic progenitor cells (45). In normal liver, CD34 is only expressed in the area around the portal vein, and most of the sinusoids in the central lobule are negative for CD34 (43). A study by Ohmori et al. discovered that there were CD34-positive but vWF-negative sinusoidal endothelial cells in the liver of patients with hepatitis C virus-related chronic liver disease, and the high expression of these CD34-positive sinusoidal endothelial cells was a risk factor for HCC carcinogenesis in these patients (46). This implies that CD34 might be a preferable endothelial marker rather than vWF for the study of HCC.

None of the pan-vascular endothelial markers mentioned above can distinguish between resting and proliferating blood vessels, while CD105 is a transmembrane glycoprotein highly and precisely expressed on activated endothelial cells (47). CD105, also known as endoglin, is a co-receptor of transforming growth factor β (TGF-β). It is involved in the development and remodeling of blood vessels, and its expression is up-regulated when resting endothelial cells become proliferative, thus representing the proliferation of hepatic sinusoidal endothelial cells in the liver (48). However, in practical application, it did not meet the expectations. Elsewhere, Qian et al. compared CD31 and CD105 in the determination of a more stable endothelial marker. The results showed that in 90 HCCs, all tumor vessels showed CD31 expression, 39 cases (43.3%) showed weak or no CD105 expression in tumors and their vessels, of which 29 cases (74.4%) were poorly differentiated HCCs, indicating that CD105 might not be expressed in poorly differentiated HCC cells (49). Evidence from Yu et al. found that CD105 was expressed in neovessels of HCC and sinusoidal endothelial cells in cirrhotic liver (50). Furthermore, given that CD105 can be expressed in tumor cells (49, 51, 52), contamination with CD105-positive tumor cells is inevitable. Therefore, the application of CD105 in the measurement of MVD in HCC is limited. CD34 is still the most widely applied endothelial marker in calculating MVD in HCC (Table 1).

**Table 1:** Measurement and prognosis of MVD in different trials.

**References**	***N***	**Marker**	**Proportion of sinusoid-like vessels**	**Counting method**	**Preoperative treatment**	**No. of hotspots**	**Correlation between high-MVD and poor prognosis**
Tanigawa et al. (53)	43	CD34 and vWF	5 out of 43	Weidner	TAE (12 cases)	5	Positive
El-assal et al. (54)	71	vWF	NM	Weidner	None	3	Positive
Sun et al. (28)	78	CD34	19 out of 78	Weidner	None	5	Irrelevant
Poon et al. (55)	100	CD34 and vWF	NM	CIAS	None	5	Positive (only for CD34 in tumor ≤ 5 cm)
Ho et al. (56)	86	CD34 and CD105	NM	Weidner	None	5	Irrelevant
Zhang et al. (57)*	82	CD34	NM	CIAS	NM	5	Positive
Yang et al. (58)	113	CD34 and CD105	NM	Weidner	None	3	Positive (only for CD105-MVD)
Sakaguchi et al. (59)	51	CD34 and CL-5	NA	Weidner	NM	5	Positive (only for CL-5-MVD)
Huang et al. (60)	100	CD34 and Endocan	NM	Weidner	None	3	Positive (only for Endocan -MVD)
Zhang et al. (61)	75	CD34	NA	Weidner	None	5	Positive
Zeng et al. (62)	69	CD34	NM	Chalkley	None	3	Negative
Kitamura et al. (63)	63	CD34	NM	CIAS	None	10	Negative
Wang et al. (64)	305	CD34	NM	CIAS	None	5	Positive
Qiu et al. (65)	103	CD34	NM	CIAS	None	NM	Positive
Murakami et al. (66)	136	CD34	20 out of 136	Weidner	None	5	Negative
Luo et al. (67)	90	CD34	NA	Weidner	None	5	Positive

N, numbers of patients; NM, not mentioned; NA, sinusoid-like vessels were measured, but the data is not available; CIAS, computerized image analysis system;

* *patients received liver transplantation*.

## Vascular Patterns that Influence MVD

**Figure 2:**
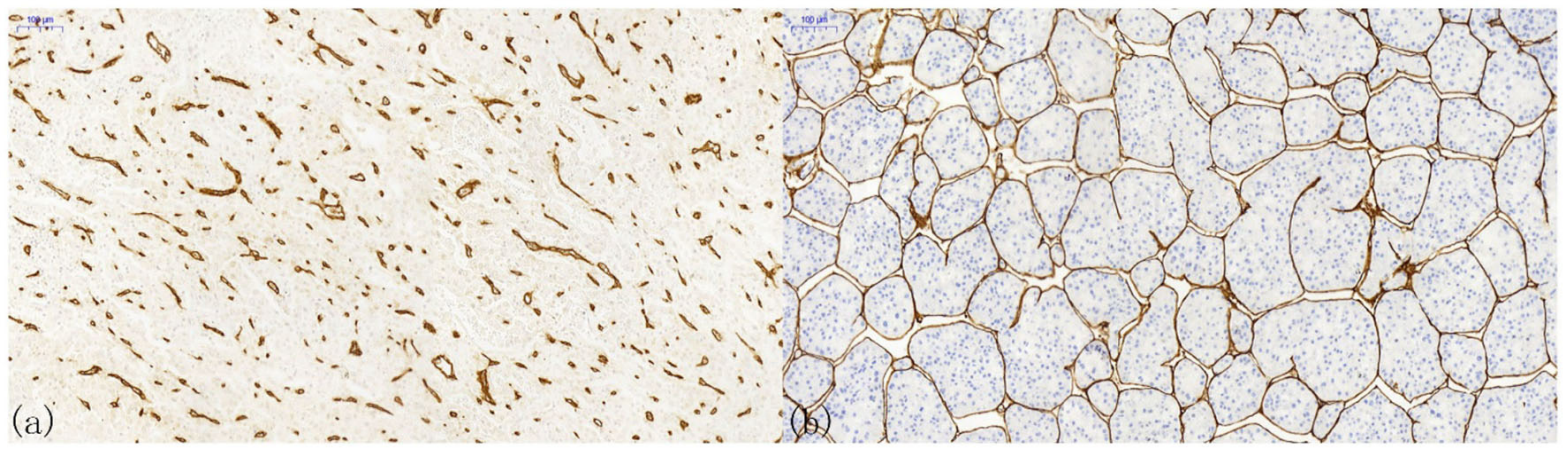
Patterns of microvessels in hepatocellular carcinoma. **(a)** Capillary-like microvessels have discrete, disconnected blood vessels with small or no lumen; **(b)** Sinusoid-like microvessels, in which the endothelial cells are interconnected, can entirely encapsulate the cancer nest to form a cobweb-like structure.

Pathological angiogenesis of HCC is often referred to as capillarization, in which normal sinusoids turn into thicker and continuous endothelial cells with fewer fenestrations (68). Two classic morphologies can ordinarily be observed in the immunohistochemical staining of HCC tissues (Figure 2). One is capillary-like microvessels, and the other is sinusoid-like microvessels (also known as vessels that encapsulate tumor clusters, VETC) (66). Generally, the former has small, scattered capillaries with no or a narrow lumen, whereas the latter has continuous branches and an apparent lumen. However, the clinicopathological differences between these two distinct vascular patterns have scarcely been observed.

The latter vascular pattern presents a hurdle to the counting of MVD in HCC. It becomes impossible to count vascular endothelial cells connected using the conventional counting principle. Therefore, Tanigawa proposed a modified counting method by defining every 40-μm lumen length as one point (53). Nonetheless, the efficacy and clinical significance of this method have not been thoroughly verified, and no one proposed a better solution after that. In addition to the measurement method being different from the conventional microvascular evaluation, sinusoidal-like vessels have a great impact on the clinical outcomes of HCC patients. An investigation by Sugino et al. checked immunohistochemical slices on 80 autopsy HCC cases and speculated that this special vascular pattern could encircle multiple tumor cells to spread in the form of multicellular tumor emboli, rather than cancer cells invading the blood vessel wall alone (69). This observation suggested an invasion-independent metastasis phenomenon in HCC. The clinical significance of this conjecture was validated by later studies. Dingand and colleagues revealed that the sinusoidal-like vascular patterns were associated with a low overall survival rate and high early recurrence rate (70). Additionally, Fang et al. unprecedentedly used three-dimensional reconstruction to confirm that sinus vessels formed an interconnected network around a single HCC nodule, while capillaries showed discrete and disorganized patterns (71, 72). They suggested that this vascular pattern was an effective mode of HCC metastasis independent of epithelial-to-mesenchymal transition. In 2019, Renne et al. demonstrated that it was an independent risk factor for early recurrence and decreased overall survival in a large multi-institutional cohort containing 541 resected HCCs from Italy, South Korea, and Japan (73). Seemingly, it reminds us that the MVD influence the clinical outcomes of HCC, and the heterogeneity of vascular patterns might regulate HCC progression.

## Prognostic Value of MVD in HCC

Tanigawa, one of the pioneers exploring the association between MVD and prognosis of HCC patients, demonstrated that patients with MVD <290 showed a better overall survival and were more likely to remain tumor-free (53). The negative correlation between MVD and prognosis of patients was confirmed in several subsequent studies and summarized in a meta-analysis (54, 55, 57–61, 64, 65, 67, 74). However, an ambiguous connection and even a positive correlation between MVD and survival in HCC patients were also observed by other investigators (28, 56, 62, 63, 66). For instance, in a retrospective study of 136 HCC patients reported by Murakami et al., low MVD was identified as an independent predictor of poor 2-year DFS and OS, which contrasted with previous findings (66). Further, other researchers tried to combine MVD with other factors in predicting the prognosis of HCC patients. Qiu and colleagues used Beclin-1 and MVD to predict survival (65), and Murakami suggested that the 10-year OS rate and 2-year DFS rate in the low vasohibin1/MVD group (vasohibin1/CD34-MVD ≤ 0.459) were significantly higher than those in the high vasohibin1/CD34-MVD group (75). These conflicting results were partly due to the influence of methodological factors including endothelial markers, hotspot selection, counting methods, microvascular patterns, and patients. For example, in the study by Murakami, sinusoidal-like microvessels were identified in about 15% of cases. This special microvascular pattern, which ranges from 18.9 to 45.2% in HCC (70, 71, 73), ordinarily has a lower blood vessel density than ordinary capillary-like pattern and tends to form a large vessel lumen (76). Importantly, it has independent negative effects on OS and DFS (69–73). Neglecting the possible impacts of microvascular pattern on prognosis will cause unpredictable interference for the judgment of the actual role of MVD. Unfortunately, the importance of microvascular patterns has been underestimated in the studies reported so far, and its subgroup analysis is largely understudied. This kind of sinus vessel occurs frequently only in HCC, thyroid carcinoma, and clear cell renal carcinoma, but it is rare in malignant tumors originating from stomach, colon, breast, pancreas, lung, uterus, and esophagus (77). Also, this might partially explain why MVD is less controversial as a prognostic factor in tumors that significantly induce angiogenesis, such as breast, prostate, and hematologic malignancies. Therefore, in analyzing the effect of MVD on tumor prognosis, the heterogeneity of microvascular patterns should be factored. In addition, differences of inclusion criteria in these trials might also have a significant impact; in particular, some subjects received preoperative radiofrequency ablation and transhepatic artery catheterization chemotherapy, while others did not. Since necrosis has a great impact on tumor angiogenesis, under the influence of such confounding factors, whether such a research could reflect the impact of MVD on tumor prognosis was highly susceptible. Therefore, it is essential to conduct a rigorously designed study where the samples are relatively uniform or with suitable layers.

Microvascular invasion (MVI) is defined as the presence of tumor cells in the endothelium-lined vascular lumen that is visible only under a microscope. Its diagnosis depends on the histological evaluation of the tumor and its surrounding liver tissue (78). In HCC, both MVI and MVD are considered to be positively correlated with earlier recurrence and shorter overall survival (79). High tumor neovascularization often represents high invasiveness. However, whether there is a correlation between MVI and high-MVD remains to be further studied. A few researchers have elaborated on this. For example, Franco et al. believed that there was a significant correlation between peri-tumor vessel invasion and MVD (80). However, the cause of invasiveness does not necessarily depend on blood vessel density. As mentioned earlier, the sinusoidal-like vascular in HCC is highly invasive. Fang et al. showed that endothelium-wrapped tumor emboli were always seen in adjacent non-tumor blood vessels (71); however, this vascular pattern is not necessarily associated with high-MVD.

## The Predictive Role of MVD in Anti-Angiogenesis Therapies

HCC tends to show vascular invasion because of its vascularization feature, and anti-angiogenic therapy is supposed to be a promising approach. Most advanced HCC treatments are currently approved in first- and second-line settings target angiogenic pathways (12). Although numerous anti-angiogenic agents have been tested or are under development, sorafenib, regorafenib, and lenvatinib are currently the only anti-angiogenic agents approved globally to enhance survival in patients with advanced HCC (81).

As the most direct indicator of angiogenesis in tumor tissue, MVD might play a vital role in regulating the efficacy of anti-angiogenic agents, but its performance was unsatisfactory. The traditional histological immunohistochemical method for measuring MVD failed in the acquisition of tumor samples greatly, which hinders its application in the evaluation of anti-angiogenic therapy. The development of a repeatable, accurate, and preferably non-invasive tumor angiogenesis evaluation technique has great clinical value in the follow-up of these targeted treatments. To overcome this, few investigators have tried to identify imaging indicators equivalent to traditional MVD for evaluating the efficacy of anti-angiogenic therapy. For example, Zhou et al. utilized contrast-enhanced ultrasound perfusion imaging to measure blood perfusion in a mouse HCC model and proved high consistency with MVD, which can be used to monitor perfusion alteration after anti-angiogenic therapy (82). Again, Song et al. used dynamic contrast-enhanced MRI to affirm if their evaluation function equals the traditional MVD (83). Recently, Lee et al. used intra-voxel incoherent motion imaging to confirm that the perfusion fraction was significantly correlated with MVD in HCC, which thus could be used to evaluate the anti-angiogenic effect of sorafenib (84). Yang and colleagues jointly introduced ultra-small superparamagnetic iron oxide enhanced susceptibility-weighted imaging (USPIO-enhanced SWI) and mean vessel density imaging into the evaluation of tumor vessels at the macro- and micro-vasculature levels, and revealed positive correlation between mean vessel density and traditional histological MVD (85). Specifically, the intra-tumoral susceptibility signal scoring in the sorafenib treatment group was significantly lower than that of control group at each time point (7 days, *P* = 0.006; 14 days, *P* = 0.013; 21 days, *P* = 0.012) (85). Previous studies could not directly demonstrate tumor microvessels, but evaluated the functional characteristics of tumor angiogenesis, while Yang's method directly reflected the changes of tumor neovascularization after treatment. This method may be useful for other anti-angiogenic chemicals in identifying those patients who might benefit from such a strategy. Despite these studies being young, clinical applications are promising. And perhaps with its advancement, MVD will guide clinical practice in a new perspective.

## Drawbacks

In addition to the special microvascular pattern in HCC, there are several points worth mentioning. First, all existing studies on microvessel density in HCC focused on inter-tumoral heterogeneity (86), but little is known about the intra-tumoral heterogeneity of angiogenesis in HCC. Recently, intra-tumoral heterogeneity of HCC has been carried out with significant differences in genomics, transcriptomics, proteomics, metabolomics, and local immunity (87, 88), but the intra-tumoral heterogeneity of neovascularization in HCC has rarely been explored, specifically, in the case of multiple lesions in the liver, which could be intrahepatic metastases of a single tumor or multi-centric carcinogenesis of several independent tumors (89). In previous investigations, the investigators normally chose only one nodular as a representative MVD measurement (62) despite three to five regions with the highest MVD being selected. Understanding the intratumoral heterogeneity of angiogenesis in HCC might help us better classify HCC and improve prognosis and antiangiogenic therapy.

Second, current indicators for identification of MVD are endothelial cell markers, however, other components of blood vessels including vascular smooth muscle cells and pericytes are not well reflected and influence the integrity of microvessels (90). It is alleged that HCC with high MVD but poor vascular quality might have worse blood perfusion, drug penetration, and more likely to metastasize than patients with low MVD but better vascular quality. Reports suggest that α-SMA was used in reflecting the maturity of microvessels in HCC, and was proved to have a certain prognostic value (64). The understanding of microvascular quality needs to be further elucidated, and this can be resolved with the discovery of novel and effective pericyte markers and the progress of imaging technology.

## Conclusions and Prospect

We reviewed the previous literature on the measurement of MVD and its roles in predicting the prognosis and reflecting the underlying evaluation of HCC. Then, we introduced a particular but not rare microvasculature pattern. With the rapid development of computer-aided technology, the measurement of MVD could facilitate automation and standardization in future applications. CD34 is still the predominant marker, however, it has been applied for 20 years. Also, the sinusoidal-like vessels in HCC might suggest clinical significance such as survival and treatment response, and its underlying biological characteristics are impressive. The intra-tumoral heterogeneity and the maturity of microvessels should be reiterated in future clinical studies, which might be helpful for the advancement of anti-angiogenesis therapy. Finally, the combination of MVD with new imaging techniques is a potential strategy for the evaluation of the treatment efficacy.

## Author Contributions

QZ and JW conceived the idea and wrote the manuscript. TL and XB supervised the study, interpreted the clinical significance, and made critical revisions to the draft. All authors contributed to the article and approved the submitted version.

## Conflict of Interest

The authors declare that the research was conducted in the absence of any commercial or financial relationships that could be construed as a potential conflict of interest.
